# Supplementary material for: Predicting resistance of clinical Abl mutations to targeted kinase inhibitors using alchemical free-energy calculations
Source: Commun Biol. 2018 Jun 13;1:70. doi: 10.1038/s42003-018-0075-x (PMC6110136; doi:10.1038/s42003-018-0075-x)
Supplement: Supplementary file 1 — Supplementary Information [file 42003_2018_75_MOESM1_ESM.pdf]

Supplementary Figures

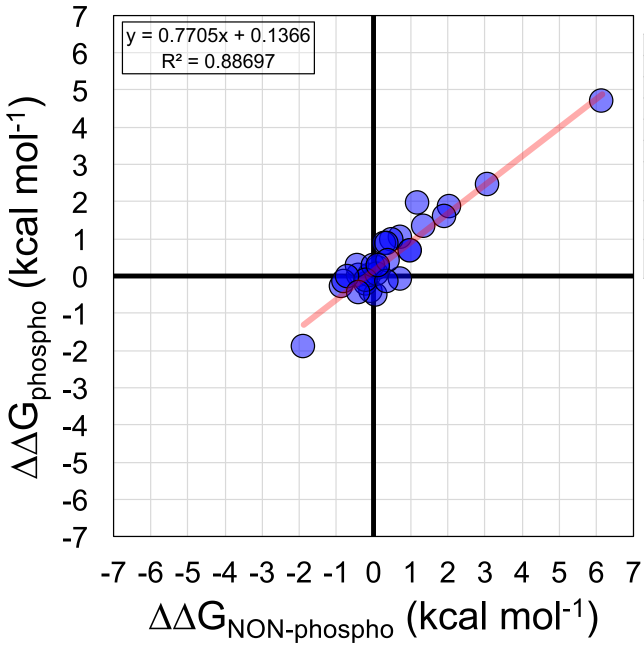

Summary Statistics

**Slope**	0.77
**Intercept** (kcal mol^{-1})	0.14
**Pearson's R**	0.94
**R²**	0.89
**RMSE** (kcal mol^{-1})	0.50
**MUE** (kcal mol^{-1})	0.39
**Accuracy (cut=1.36** kcal mol^{-1}**)**	0.94
**Specificity (cut=1.36** kcal mol^{-1}**)**	0.96
**Sensitivity (cut=1.36** kcal mol^{-1}**)**	0.80

Raw Data (adapted de Davis et al.)

TKI	ddG_NON-P (kcal mol^{-1})	ddG_phos (kcal mol^{-1})
axitinib	2.05	1.85
	1.37	1.32
	-0.86	-0.27
	0.74	1.02
	-1.88	-1.89
bosutinib	0.99	0.69
	-0.05	-0.40
	-0.39	0.00
	-0.20	-0.23
	3.08	2.47
dasatinib	0.74	-0.07
	0.06	-0.53
	-0.09	0.00
	0.15	0.20
	6.16	4.69
imatinib	1.20	1.98
	0.49	0.99
	1.00	0.67
	0.29	0.88
	**ND**	**ND**
nilotinib	0.35	0.87
	0.11	0.04
	-0.43	0.29
	0.00	0.29
	2.50	**ND**
gefitinib	1.92	1.59
	0.39	0.41
	-0.78	-0.16
	-0.22	-0.13
	0.38	-0.15
erlotinib	**ND**	1.36
	0.12	0.29
	-0.70	-0.03
	-0.41	-0.44
	**ND**	**ND**

Supplementary Figure 1. Comparison of 31 mutations for which phosphorylated and non-phosphorylated ΔK_ds were available. Scatter plot compares ΔΔGs (derived from the ΔK_ds) and contains the best-fit line with slope 0.77 and intercept 0.14. Summary statistics for this comparison are also shown. The raw ΔΔGs used for this comparison were adapted from [1]; kino-bead data for ponatinib was not available. **ND**: No data. **TKI**: Targeted kinase inhibitor.

cutoff	Experiment	Prime 0.95 kcal/mol: Prediction s	Prime 0.95 kcal/mol: Prediction r	Prime 1.77 kcal/mol: Prediction s	Prime 1.77 kcal/mol: Prediction r	Prime 2.72 kcal/mol: Prediction s	Prime 2.72 kcal/mol: Prediction r	FEP+ 0.95 kcal/mol: Prediction s	FEP+ 0.95 kcal/mol: Prediction r	FEP+ 1.77 kcal/mol: Prediction s	FEP+ 1.77 kcal/mol: Prediction r	FEP+ 2.72 kcal/mol: Prediction s	FEP+ 2.72 kcal/mol: Prediction r
axitinib	S	20	6	23	3	24	2	20	6	23	3	26	0
	R	0	0	0	0	0	0	0	0	0	0	0	0
bosutinib	S	13	4	16	4	16	5	13	4	18	1	21	0
	R	1	3	0	1	0	0	2	2	2	0	0	0
dasatinib	S	11	2	14	2	17	3	11	2	16	0	19	1
	R	2	6	1	4	1	0	3	5	1	4	0	1
imatinib	S	7	7	10	7	18	2	13	1	17	0	19	1
	R	5	2	3	1	0	0	4	3	2	2	0	0
nilotinib	S	8	8	11	7	14	6	10	6	18	0	20	0
	R	4	1	2	1	0	1	3	2	1	2	0	1
ponatinib	S	14	6	18	3	21	0	17	3	21	0	21	0
	R	1	0	0	0	0	0	1	0	0	0	0	0

Supplementary Figure 2. TKI-by-TKI truth tables with increasingly large classification cutoffs. Truth tables for the six TKIs (axitinib, bosutinib, dasatinib, imatinib, nilotinib, and ponatinib) using Prime (left, green) and FEP+ (right, blue) with classification cutoff values defining whether mutations are susceptible (S, experiment; s, prediction) or resistant (R, experiment; r, prediction). A mutation is susceptible if $\Delta\Delta G \leq$ **cutoff** or resistant if $\Delta\Delta G >$ **cutoff**.

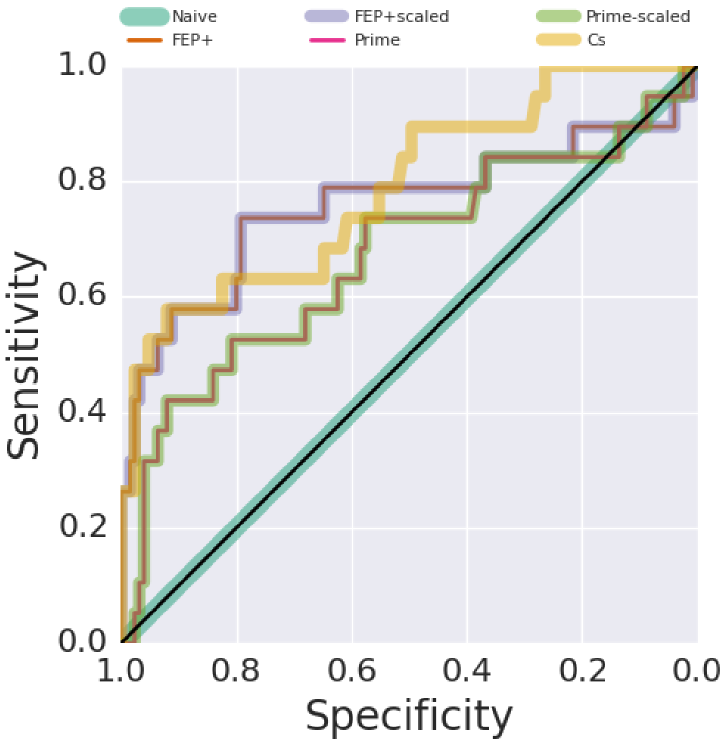

Supplementary Figure 3. ROC curves for non-scaled and scaled FEP+, non-scaled and scaled Prime, a consensus model and a naïve model. ROC-AUC for scaled and non-scaled FEP+ was $0.75^{0.90}_{0.61}$ (n=144); ROC-AUC for scaled and non-scaled Prime was $0.66^{0.81}_{0.52}$ (n=144); ROC-AUCs for the naïve model and consensus model were $0.50^{0.50}_{0.50}$ (n=144) and $0.78^{0.90}_{0.67}$ (n=144) respectively. Optimal scaling factors (a=0.34 for FEP+; a=0.23 for Prime) obtained using linear regression (m=142) were applied to the full dataset (n=144), which was used in this ROC analysis. ROC-AUC interpretations: [0.50,0.60], failure; [0.60,0.70], poor; [0.70,0.80], fair; [0.80,0.90], good; [0.90,1.00], excellent.

Supplementary Tables

Supplementary Table 1. $\Delta\Delta$G data derived from publicly available ΔpIC$_{50}$ measurements and sources of mutation clinical-observation

Mutation	axitinib $\Delta\Delta$G (kcal/mol)	bosutinib $\Delta\Delta$G (kcal/mol)	dasatinib $\Delta\Delta$G (kcal/mol)	imatinib $\Delta\Delta$G (kcal/mol)	nilotinib $\Delta\Delta$G (kcal/mol)	ponatinib $\Delta\Delta$G (kcal/mol)	gefitinib $\Delta\Delta$G (kcal/mol)	erlotinib $\Delta\Delta$G (kcal/mol)	Source of Clinical-Observation
M244V	-0.11	0.43	0.00	0.21	-0.13	0.00	nd	nd	A
L248R	0.31	1.50	0.65	2.33	2.15	0.58	nd	nd	B
L248V	0.32	0.56	0.55	0.64	0.33	0.17	nd	nd	A,C
G250E	0.27	0.11	0.41	1.01	0.60	0.30	nd	nd	A,C,D
Q252H	0.20	nd	nd	nd	nd	nd	-0.44	-0.13	A
Y253F	0.26	-0.34	0.24	1.90	1.48	0.30	-0.17	0.00	C
Y253H	0.03	nd	nd	nd	nd	nd	nd	nd	A,C,D
E255K	0.26	0.56	0.90	1.50	1.27	0.41	-0.11	-0.11	A,C,D
E255V	0.30	0.66	1.02	2.22	2.36	1.00	nd	nd	A,C
D276G	0.18	nd	nd	nd	nd	nd	nd	nd	C
E279K	-0.03	nd	nd	nd	nd	nd	nd	nd	C
E292L	0.03	nd	nd	nd	nd	nd	nd	nd	E
V299L	-0.88	1.70	1.24	0.23	0.28	0.17	nd	nd	C
T315A	-0.45	0.32	2.02	0.51	0.72	0.17	nd	nd	C
T315I	-1.27	2.45	5.08	2.32	3.75	0.41	nd	-0.15	C,D
T315V	-1.73	nd	nd	nd	nd	nd	nd	nd	B
F317C	nd	0.50	1.86	0.28	0.04	0.00	nd	nd	A[g]
F317I	nd	0.71	1.79	0.17	0.30	0.51	1.35	1.58	C
F317L	0.23	0.09	0.96	0.72	0.20	0.17	0.29	0.40	C,D
F317R	0.27	nd	nd	nd	nd	nd	nd	nd	B
F317V	0.28	1.72	2.36	0.97	0.33	0.72	nd	nd	C
M343T	0.21	nd	nd	nd	nd	nd	nd	nd	F[h]
M351T	-0.24	0.19	0.00	0.42	0.00	0.17	0.05	-0.08	A,C,D
E355A	nd	0.02	0.24	0.47	0.11	0.51	nd	nd	C
F359C	nd	-0.01	0.00	0.77	0.68	0.41	nd	nd	C
F359I	0.10	0.04	0.24	0.28	0.86	0.77	nd	nd	A
F359V	0.07	-0.11	0.00	0.32	0.60	0.17	nd	nd	A,C
L384M	0.06	nd	nd	nd	nd	nd	nd	nd	F[i]
H396R	0.25	-0.10	0.00	0.40	0.25	0.17	nd	nd	A[j]
F486S	0.05	nd	nd	nd	nd	nd	nd	nd	A[k]
E459K	nd	0.35	0.41	0.66	0.55	0.30	nd	nd	C

A: Gruber et al. ([2]), *B*: Redaelli et al. ([3]), *C*: Cortes et al. ([4]), *D*: Branford et al. ([5]), *E*: Press et al. ([6]), *F*: Shah et al. ([7]), [g] F317C observed with Δ27-183, [h] M343T observed as compound mutation with H396R, [i] L384M observed as compound mutation with M343T, [j] H396R observed as compound mutation with F486S, [k] F486S observed as compound mutation with H396R

Supplementary Table 2. Axitinib: experimental IC_{50} values and alchemical free-energy $\Delta\Delta$Gs for each mutation

	Expt. IC_{50} (nM)	Expt. ΔΔG (kcal/mol)	Prime ΔΔG (kcal/mol)	FEP+$_{Run1}$ ΔΔG (kcal/mol)	FEP+$_{Run1}$ BAR err (kcal/mol)	FEP+$_{Run2}$ ΔΔG (kcal/mol)	FEP+$_{Run2}$ BAR err (kcal/mol)	FEP+$_{Run3}$ ΔΔG (kcal/mol)	FEP+$_{Run3}$ BAR err (kcal/mol)	$\Delta\Delta G_{Av}$ (kcal/mol)	SE (kcal/mol)
wild-type	823										
M244V	690	-0.11	-0.10	-0.40	0.41	-0.35	0.41	-0.43	0.41	-0.39	0.02
L248R	1393	0.31	-0.06	2.13	0.43	2.42	0.45	2.46	0.43	2.34	0.10
L248V	1399	0.32	6.02	-1.32	0.41	-1.04	0.42	-1.22	0.42	-1.19	0.08
G250E	1295	0.27	0.31	-0.35	0.41	-0.71	0.41	-0.74	0.41	-0.60	0.13
Q252H	1155	0.20	-0.18	0.07	0.43	0.30	0.42	0.29	0.43	0.22	0.08
Y253F	1275	0.26	1.11	0.77	0.43	0.23	0.43	1.15	0.45	0.72	0.27
Y253H	867	0.03	4.65	1.14	0.47	0.38	0.49	-0.19	0.45	0.44	0.39
E255K	1282	0.26	0.12	1.30	0.44	0.63	0.43	1.10	0.44	1.01	0.20
E255V	1350	0.30	-0.29	0.98	0.42	1.04	0.42	1.26	0.43	1.09	0.09
D276G	1105	0.18	-0.01	0.03	0.42	0.64	0.42	0.44	0.43	0.37	0.18
E279K	778	-0.03	-0.15	0.06	0.42	-0.22	0.43	1.27	0.43	0.37	0.46
E292L	863	0.03	-0.00	0.53	0.43	0.35	0.42	0.31	0.42	0.40	0.07
V299L	188	-0.88	-5.00	-1.08	0.42	-1.39	0.42	-1.37	0.42	-1.28	0.10
T315A	389	-0.45	0.99	0.09	0.43	0.24	0.47	0.31	0.42	0.21	0.06
T315I	98	-1.27	-2.30	-1.26	0.42	-1.50	0.45	-1.39	0.43	-1.38	0.07
T315V	45	-1.73	-1.07	-1.10	0.41	-1.32	0.42	-1.15	0.48	-1.19	0.07
F317L	1220	0.23	1.29	-0.64	0.41	-0.10	0.41	-0.38	0.41	-0.37	0.16
F317R	1286	0.27	-2.46	2.64	0.46	2.27	0.51	1.38	0.47	2.10	0.37
F317V	1320	0.28	2.29	0.45	0.42	0.70	0.42	0.75	0.42	0.63	0.09
M343T	1175	0.21	-0.04	-0.26	0.54	-0.50	0.53	-0.58	0.50	-0.45	0.10
M351T	553	-0.24	-0.07	-0.25	0.41	-0.03	0.41	0.37	0.41	0.03	0.18
F359I	975	0.10	-0.04	1.89	0.41	1.60	0.42	1.78	0.41	1.76	0.08
F359V	933	0.07	-0.07	2.68	0.42	1.55	0.42	1.64	0.41	1.96	0.36
L384M	916	0.06	-0.01	-0.07	0.41	0.27	0.41	0.23	0.41	0.14	0.11
H396R	1247	0.25	-0.02	0.36	0.42	1.23	0.41	0.65	0.42	0.75	0.26
F486S	897	0.05	-0.09	0.65	0.47	1.14	0.46	0.44	0.48	0.74	0.21

BAR err: Bennett Acceptance Ratio error.
$\Delta\Delta G_{Av}$: Average of three independent FEP+ runs.
SE: Standard Error between three independent FEP+ runs.

Supplementary Table 3. Bosutinib: experimental IC_{50} values and alchemical free-energy ΔΔGs for each mutation

	Expt. IC_{50} (nM)	Expt. ΔΔG (kcal/mol)	Prime ΔΔG (kcal/mol)	$FEP+_{Run1}$ ΔΔG (kcal/mol)	$FEP+_{Run1}$ BAR err (kcal/mol)	$FEP+_{Run2}$ ΔΔG (kcal/mol)	$FEP+_{Run2}$ BAR err (kcal/mol)	$FEP+_{Run3}$ ΔΔG (kcal/mol)	$FEP+_{Run3}$ BAR err (kcal/mol)	$ΔΔG_{Av}$ (kcal/mol)	SE (kcal/mol)
wild-type	71										
M244V	147	0.43	0.02	-0.28	0.41	-0.11	0.41	-0.08	0.41	-0.16	0.06
L248R	874	1.50	3.67	1.00	0.43	1.63	0.43	1.33	0.43	1.32	0.18
L248V	182	0.56	5.77	0.37	0.41	0.72	0.42	0.38	0.42	0.49	0.12
G250E	85	0.11	-0.30	0.28	0.43	0.63	0.43	-1.07	0.43	-0.05	0.52
Y253F	40	-0.34	-0.03	0.21	0.45	0.02	0.43	0.95	0.43	0.39	0.28
E255K	181	0.56	0.49	-1.01	0.43	-1.30	0.43	-1.01	0.43	-1.11	0.10
E255V	214	0.66	0.11	-0.47	0.42	-0.51	0.43	-0.91	0.43	-0.63	0.14
V299L	1228	1.70	-0.85	0.97	0.43	0.90	0.42	0.85	0.42	0.91	0.03
T315A	122	0.32	1.00	-1.61	0.41	-1.61	0.41	-1.97	0.41	-1.73	0.12
T315I	4338	2.45	3.75	-2.32	0.43	-2.21	0.42	-1.26	0.42	-1.93	0.34
F317C	165	0.50	4.83	1.04	0.41	1.27	0.41	1.22	0.42	1.18	0.07
F317I	232	0.71	1.61	0.16	0.41	0.07	0.42	0.02	0.41	0.08	0.04
F317L	82	0.09	-0.71	0.05	0.41	0.47	0.41	0.24	0.41	0.25	0.12
F317V	1280	1.72	4.12	1.98	0.42	1.50	0.42	2.25	0.42	1.91	0.22
M351T	97	0.19	0.02	0.36	0.42	0.82	0.41	0.71	0.41	0.63	0.14
E355A	74	0.02	0.13	-0.20	0.44	0.13	0.43	0.27	0.43	0.07	0.14
F359C	70	-0.01	-0.09	3.02	0.42	2.51	0.42	1.97	0.43	2.50	0.30
F359I	76	0.04	-0.06	0.66	0.41	1.74	0.41	1.43	0.42	1.28	0.32
F359V	59	-0.11	-0.06	0.98	0.43	1.69	0.41	1.91	0.42	1.53	0.28
H396R	60	-0.10	-1.07	0.62	0.42	-0.07	0.42	-0.93	0.43	-0.13	0.45
E459K	127	0.35	0.26	-0.69	0.42	0.23	0.42	-0.54	0.42	-0.33	0.28

BAR err: Bennett Acceptance Ratio error.
$ΔΔG_{Av}$: Average of three independent FEP+ runs.
SE: Standard Error between three independent FEP+ runs.

Supplementary Table 4. Dasatinib: experimental IC_{50} values and alchemical free-energy ΔΔGs for each mutation

	Expt. IC_{50} (nM)	Expt. ΔΔG (kcal/mol)	Prime ΔΔG (kcal/mol)	$FEP+_{Run1}$ ΔΔG (kcal/mol)	$FEP+_{Run1}$ BAR err (kcal/mol)	$FEP+_{Run2}$ ΔΔG (kcal/mol)	$FEP+_{Run2}$ BAR err (kcal/mol)	$FEP+_{Run3}$ ΔΔG (kcal/mol)	$FEP+_{Run3}$ BAR err (kcal/mol)	$ΔΔG_{Av}$ (kcal/mol)	SE (kcal/mol)
wild-type	2										
M244V	2	0.00	-0.10	0.05	0.41	-0.37	0.41	-0.43	0.41	-0.25	0.15
L248R	6	0.65	-2.13	1.40	0.42	1.50	0.43	1.51	0.42	1.47	0.04
L248V	5	0.55	2.60	0.58	0.42	0.70	0.41	0.79	0.41	0.69	0.06
G250E	4	0.41	-0.00	-0.54	0.43	-0.31	0.43	0.01	0.44	-0.28	0.16
Y253F	3	0.24	0.00	-0.21	0.43	-0.24	0.43	-0.03	0.44	-0.16	0.07
E255K	9	0.90	-0.08	-0.30	0.43	-0.17	0.44	-1.05	0.43	-0.51	0.27
E255V	11	1.02	-0.08	0.06	0.42	-0.80	0.42	-0.12	0.42	-0.29	0.26
V299L	16	1.24	0.01	0.83	0.41	0.36	0.42	0.77	0.42	0.65	0.15
T315A	59	2.02	5.09	-1.74	0.41	-1.65	0.41	-1.23	0.41	-1.54	0.16
T315I	10000	5.08	-2.69	5.63	0.43	4.69	0.44	5.50	0.43	5.27	0.29
F317C	45	1.86	4.72	2.63	0.42	2.32	0.42	2.62	0.41	2.52	0.10
F317I	40	1.79	2.38	1.94	0.41	2.04	0.41	1.94	0.41	1.97	0.03
F317L	10	0.96	1.22	1.26	0.41	1.42	0.41	1.08	0.41	1.25	0.10
F317V	104	2.36	4.08	3.12	0.42	2.84	0.42	2.68	0.42	2.88	0.13
M351T	2	0.00	0.04	0.04	0.41	0.14	0.41	0.00	0.42	0.06	0.04
E355A	3	0.24	0.00	-0.24	0.43	-0.87	0.45	-1.25	0.44	-0.79	0.29
F359C	2	0.00	-0.03	1.24	0.42	0.68	0.41	1.38	0.42	1.10	0.21
F359I	3	0.24	-0.02	-0.50	0.42	-0.33	0.42	-1.14	0.42	-0.66	0.25
F359V	2	0.00	-0.03	-0.87	0.41	0.57	0.42	-0.62	0.41	-0.31	0.44
H396R	2	0.00	2.53	-0.76	0.43	-0.09	0.43	-0.06	0.43	-0.30	0.23
E459K	4	0.41	0.00	-0.68	0.42	-0.17	0.42	-0.07	0.41	-0.31	0.19

T315I was beyond the concentration limit of the assay (10,000 nM).
BAR err: Bennett Acceptance Ratio error.
$ΔΔG_{Av}$: Average of three independent FEP+ runs.
SE: Standard Error between three independent FEP+ runs.

Supplementary Table 5. Imatinib: experimental IC_{50} values and alchemical free-energy ΔΔGs for each mutation

	Expt. IC_{50} (nM)	Expt. ΔΔG (kcal/mol)	Prime ΔΔG (kcal/mol)	FEP+$_{Run1}$ ΔΔG (kcal/mol)	FEP+$_{Run1}$ BAR err (kcal/mol)	FEP+$_{Run2}$ ΔΔG (kcal/mol)	FEP+$_{Run2}$ BAR err (kcal/mol)	FEP+$_{Run3}$ ΔΔG (kcal/mol)	FEP+$_{Run3}$ BAR err (kcal/mol)	ΔΔG$_{Av}$ (kcal/mol)	SE (kcal/mol)
wild-type	201										
M244V	287	0.21	-0.08	0.15	0.41	0.43	0.41	0.17	0.41	0.25	0.09
L248R	10000	2.33	1.92	1.92	0.43	2.52	0.44	2.34	0.43	2.26	0.18
L248V	586	0.64	1.89	-1.04	0.41	-1.02	0.42	-1.20	0.41	-1.09	0.06
G250E	1087	1.01	0.92	0.16	0.41	0.02	0.41	0.12	0.41	0.10	0.04
Y253F	4908	1.90	-0.02	0.87	0.43	0.65	0.42	1.34	0.44	0.95	0.20
E255K	2487	1.50	0.25	-0.12	0.44	1.95	0.44	-0.55	0.44	0.43	0.77
E255V	8322	2.22	0.24	-0.72	0.42	-0.02	0.42	-0.53	0.43	-0.42	0.21
V299L	295	0.23	-1.29	0.66	0.41	0.26	0.42	-0.37	0.42	0.18	0.30
T315A	476	0.51	5.10	-1.39	0.41	-1.86	0.41	-2.09	0.44	-1.78	0.21
T315I	9773	2.32	0.88	4.23	0.43	4.23	0.42	3.14	0.44	3.87	0.36
F317C	324	0.28	2.10	0.27	0.42	-0.18	0.41	0.45	0.42	0.18	0.19
F317I	266	0.17	0.94	0.59	0.41	0.66	0.41	0.48	0.41	0.58	0.05
F317L	675	0.72	0.74	0.58	0.41	0.53	0.41	0.38	0.41	0.50	0.06
F317V	1023	0.97	1.57	0.71	0.42	0.79	0.42	0.80	0.41	0.77	0.03
M351T	404	0.42	-0.02	1.72	0.41	1.03	0.42	1.20	0.42	1.32	0.21
E355A	441	0.47	0.29	0.13	0.43	0.08	0.44	0.14	0.43	0.12	0.02
F359C	728	0.77	2.43	0.88	0.42	0.47	0.41	0.33	0.42	0.56	0.17
F359I	324	0.28	1.95	-0.13	0.41	-0.87	0.41	0.08	0.41	-0.31	0.29
F359V	346	0.32	2.53	-0.66	0.41	0.02	0.41	-0.27	0.42	-0.30	0.20
H396R	395	0.40	2.76	-0.39	0.41	-0.38	0.42	-0.39	0.42	-0.39	0.00
E459K	612	0.66	0.24	-0.09	0.43	-0.09	0.42	-0.08	0.42	-0.09	0.00

T315I was beyond the concentration limit of the assay (10,000 nM).
BAR err: Bennett Acceptance Ratio error.
ΔΔG$_{Av}$: Average of three independent FEP+ runs.
SE: Standard Error between three independent FEP+ runs.

Supplementary Table 6. Nilotinib: experimental IC_{50} values and alchemical free-energy ΔΔGs for each mutation

	Expt. IC_{50} (nM)	Expt. ΔΔG (kcal/mol)	Prime ΔΔG (kcal/mol)	FEP+$_{Run1}$ ΔΔG (kcal/mol)	FEP+$_{Run1}$ BAR err (kcal/mol)	FEP+$_{Run2}$ ΔΔG (kcal/mol)	FEP+$_{Run2}$ BAR err (kcal/mol)	FEP+$_{Run3}$ ΔΔG (kcal/mol)	FEP+$_{Run3}$ BAR err (kcal/mol)	ΔΔG_{Av} (kcal/mol)	SE (kcal/mol)
wild-type	15										
M244V	12	-0.13	-0.11	0.15	0.41	-0.21	0.41	0.21	0.41	0.05	0.13
L248R	549	2.15	0.48	2.05	0.43	2.12	0.47	1.93	0.43	2.03	0.06
L248V	26	0.33	3.53	-0.50	0.42	-0.39	0.41	-0.92	0.41	-0.60	0.16
G250E	41	0.60	0.05	0.06	0.41	-0.27	0.41	-0.38	0.41	-0.20	0.13
Y253F	179	1.48	-0.27	1.09	0.43	0.42	0.42	1.16	0.42	0.89	0.24
E255K	127	1.27	0.41	-2.24	0.48	-1.52	0.46	0.33	0.46	-1.14	0.77
E255V	784	2.36	-0.03	0.31	0.42	-0.25	0.43	-0.55	0.43	-0.16	0.25
V299L	24	0.28	2.94	-0.18	0.41	0.21	0.41	0.15	0.41	0.06	0.12
T315A	50	0.72	3.38	-1.33	0.41	-1.31	0.41	-1.39	0.41	-1.34	0.02
T315I	8091	3.75	4.16	4.29	0.43	5.00	0.42	4.34	0.43	4.54	0.23
F317C	16	0.04	0.90	1.34	0.41	0.88	0.41	0.60	0.41	0.94	0.22
F317I	25	0.30	-0.18	1.24	0.41	1.17	0.41	0.82	0.41	1.08	0.13
F317L	21	0.20	1.74	1.03	0.41	1.07	0.41	1.09	0.41	1.06	0.02
F317V	26	0.33	0.77	1.16	0.41	0.68	0.42	1.07	0.42	0.97	0.15
M351T	15	0.00	0.09	-0.06	0.41	-0.09	0.42	-0.46	0.42	-0.20	0.13
E355A	18	0.11	-0.06	-0.46	0.43	-1.01	0.43	-0.32	0.43	-0.60	0.21
F359C	47	0.68	3.68	1.32	0.41	1.44	0.41	1.52	0.41	1.43	0.06
F359I	64	0.86	3.70	1.05	0.41	1.13	0.41	0.74	0.41	0.97	0.12
F359V	41	0.60	3.67	1.00	0.41	1.08	0.41	1.38	0.42	1.15	0.12
H396R	23	0.25	2.58	-0.07	0.42	0.21	0.42	0.03	0.42	0.06	0.08
E459K	38	0.55	-0.00	-0.17	0.42	-0.46	0.42	-0.10	0.42	-0.24	0.11

BAR err: Bennett Acceptance Ratio error.
ΔΔG_{Av}: Average of three independent FEP+ runs.
SE: Standard Error between three independent FEP+ runs.

Supplementary Table 7. Ponatinib: experimental IC_{50} values and alchemical free-energy ΔΔGs for each mutation

	Expt. IC_{50} (nM)	Expt. ΔΔG (kcal/mol)	Prime ΔΔG (kcal/mol)	FEP+$_{Run1}$ ΔΔG (kcal/mol)	FEP+$_{Run1}$ BAR err (kcal/mol)	FEP+$_{Run2}$ ΔΔG (kcal/mol)	FEP+$_{Run2}$ BAR err (kcal/mol)	FEP+$_{Run3}$ ΔΔG (kcal/mol)	FEP+$_{Run3}$ BAR err (kcal/mol)	ΔΔG$_{Av}$ (kcal/mol)	SE (kcal/mol)
wild-type	3										
M244V	3	0.00	-0.13	0.07	0.41	-0.28	0.41	0.12	0.41	-0.03	0.13
L248R	8	0.58	2.48	1.40	0.43	0.96	0.43	1.10	0.44	1.15	0.13
L248V	4	0.17	2.48	-1.82	0.42	-1.23	0.42	-1.96	0.42	-1.67	0.22
G250E	0.021	0.30	0.17	-0.32	0.43	-0.25	0.43	-0.71	0.46	-0.43	0.14
Y253F	5	0.30	0.05	0.85	0.43	1.32	0.44	0.77	0.43	0.98	0.17
E255K	6	0.41	1.05	-0.27	0.48	-0.66	0.48	0.03	0.47	-0.30	0.20
E255V	16	1.00	-0.04	1.19	0.43	0.94	0.43	-0.41	0.43	0.57	0.50
V299L	4	0.17	-0.29	-0.56	0.41	-0.55	0.41	-1.42	0.41	-0.84	0.29
T315A	4	0.17	-0.51	-2.90	0.41	-3.15	0.41	-2.92	0.41	-2.99	0.08
T315I	6	0.41	-5.42	0.51	0.42	0.90	0.42	0.91	0.42	0.77	0.13
F317C	3	0.00	1.45	0.44	0.41	0.98	0.42	0.80	0.41	0.74	0.16
F317I	7	0.51	0.62	-0.76	0.41	-1.03	0.41	-1.02	0.41	-0.94	0.09
F317L	4	0.17	0.57	-1.08	0.41	-0.83	0.41	-0.85	0.41	-0.92	0.08
F317V	10	0.72	1.14	0.05	0.41	-0.21	0.41	0.24	0.42	0.03	0.13
M351T	4	0.17	-0.12	0.89	0.41	1.66	0.41	0.65	0.41	1.07	0.30
E355A	7	0.51	0.01	0.12	0.44	-0.52	0.44	-0.55	0.43	-0.32	0.22
F359C	6	0.41	2.12	0.25	0.42	-0.35	0.43	0.73	0.42	0.21	0.31
F359I	11	0.77	0.34	-0.66	0.41	-0.38	0.41	0.06	0.41	-0.33	0.21
F359V	4	0.17	0.74	0.11	0.41	-0.28	0.41	0.08	0.42	-0.03	0.13
H396R	4	0.17	-0.04	0.19	0.49	0.10	0.45	-1.41	0.48	-0.37	0.52
E459K	5	0.30	-0.00	-0.51	0.42	-0.78	0.42	-0.63	0.42	-0.64	0.08

BAR err: Bennett Acceptance Ratio error.
ΔΔG$_{Av}$: Average of three independent FEP+ runs.
SE: Standard Error between three independent FEP+ runs.

Supplementary Table 8. Summary of statistics of scaled predictions, a naïve model, and a consensus model

Method	Scaling factor	MUE (kcal/mol)	RMSE (kcal/mol)	Accuracy	Specificity	Sensitivity
		[N=142]	[N=142]	[N=144]	[N=144]	[N=144]
Prime	1.00	$1.14^{1.35}_{0.94}$	$1.70^{1.97}_{1.40}$	$0.73^{0.80}_{0.65}$	$0.76^{0.83}_{0.68}$	$0.53^{0.78}_{0.29}$
Prime	0.50	$0.64^{0.76}_{0.53}$	$0.91^{1.06}_{0.77}$	$0.84^{0.90}_{0.78}$	$0.90^{0.95}_{0.84}$	$0.42^{0.65}_{0.20}$
Prime	0.33	$0.53^{0.62}_{0.44}$	$0.76^{0.87}_{0.63}$	$0.87^{0.92}_{0.81}$	$0.96^{0.99}_{0.92}$	$0.26^{0.47}_{0.08}$
Prime	0.23	$0.49^{0.59}_{0.40}$	$0.73^{0.86}_{0.60}$	$0.86^{0.92}_{0.81}$	$0.99^{1.00}_{0.97}$	$0.00^{0.00}_{0.00}$
FEP+	1.00	$0.79^{0.91}_{0.67}$	$1.07^{1.27}_{0.89}$	$0.88^{0.93}_{0.81}$	$0.94^{0.98}_{0.89}$	$0.47^{0.72}_{0.22}$
FEP+	0.34	$0.55^{0.64}_{0.47}$	$0.78^{0.91}_{0.65}$	$0.88^{0.93}_{0.83}$	$1.00^{1.00}_{1.00}$	$0.11^{0.27}_{0.00}$
Naive	—	$0.57^{0.69}_{0.46}$	$0.87^{1.04}_{0.70}$	$0.87^{0.92}_{0.81}$	$1.00^{1.00}_{1.00}$	$0.00^{0.00}_{0.00}$
Consensus	—	$0.47^{0.56}_{0.39}$	$0.71^{0.84}_{0.59}$	$0.87^{0.92}_{0.81}$	$1.00^{1.00}_{1.00}$	$0.00^{0.00}_{0.00}$

Supplementary Table 9. Summary of the preparation of the 6 Abl:TKI co-crystal structure complexes

				Experimental structure				Prepared model used for simulations							
**PDB**	**Receptor**	**Ligand**	**Chains**	**# Water**[a]	**# Rec. atoms,** (Chain) [b]	**# Aminos,** (Chain)	**Chain** used	**# Water**	**# Rec.** atoms	**# Rec.** aminos	**# Ash**	**# Glh**	**# Hip**	**# Lig.** atoms	**Het. atom**[d] w/ proton
4wa9	Abl	Axit	A, B	305	2219 (B)	276 (B)	B	131	4580	284	Ash421	0	0	46	neutral
3ue4	Abl	Bosut	A, B	152	2187 (A)	270 (A)	A	89	4581	284	Ash421 Ash381	0	0	66	NBI,4401
4xey	Abl	Dasat	A, B	0	2195 (A)	269 (A)	A	0	4581	284	Ash421[c] Ash381[c]	0	0	59	neutral
1opj	Abl	Imat	A, B	231	2336 (B)	288 (B)	B	104	4579	284	0	0	0	69	N51,4767
3cs9	Abl	Nilot	A, B, C, D	266	2142 (A)	264 (A)	A	99	4579	284	0	0	0	61	neutral
3oxz	Abl	Ponat	A	89	2152 (A)	268 (A)	A	89	4580	284	0	0	0	67	N3,2155

[a]Total number of water molecules, [b]Count includes N-Acetyl/N-terminal (6 atoms) and N-methylamide/C-terminal (6 atoms) capping groups, [c]Original index in experimental structure was Ash440, Ash400, [d](PDB atom name) and (PDB atom serial number).
Ash: Neutral form of Asp; **Glh**: Neutral form of Glu; **Hip**: Charged form of His.

Supplementary References

[1] Davis, M. I. *et al.* Comprehensive analysis of kinase inhibitor selectivity. *Nat. Biotechnol.* **29**, 1046–1051 (2011).

[2] Gruber, F. X. *et al.* BCR-ABL isoforms associated with intrinsic or acquired resistance to imatinib: More heterogeneous than just ABL kinase domain point mutations? *Med. Oncol.* **29**, 219–226 (2012).

[3] Redaelli, S. *et al.* Three novel patient-derived bcr/abl mutants show different sensitivity to second and third generation tyrosine kinase inhibitors. *Ame. J. Hematol.* **87**, E125–E128 (2012).

[4] Cortes, J. E. *et al.* Ponatinib in Refractory Philadelphia Chromosome–Positive Leukemias. *N. Engl. J. Med.* **367**, 2075–2088 (2012).

[5] Branford, S. High frequency of point mutations clustered within the adenosine triphosphate-binding region of BCR/ABL in patients with chronic myeloid leukemia or Ph-positive acute lymphoblastic leukemia who develop imatinib (STI571) resistance. *Blood* **99**, 3472–3475 (2002).

[6] Press, R. D., Willis, S. G., Laudadio, J., Mauro, M. J. & Deininger, M. W. N. Determining the rise in bcr-abl rna that optimally predicts a kinase domain mutation in patients with chronic myeloid leukemia on imatinib. *Blood* **114**, 2598–2605 (2009).

[7] Shah, N. P. *et al.* Multiple BCR-ABL kinase domain mutations confer polyclonal resistance to the tyrosine kinase inhibitor imatinib (STI571) in chronic phase and blast crisis chronic myeloid leukemia. *Cancer Cell* **2**, 117–125 (2002).
