# Supplementary material for: Predicting resistance of clinical Abl mutations to targeted kinase inhibitors using alchemical free-energy calculations
Source: Commun Biol. 2018 Jun 13;1:70. doi: 10.1038/s42003-018-0075-x (PMC6110136; doi:10.1038/s42003-018-0075-x)
Supplement: Supplementary file 2 — Description of Additional Supplementary Files [file 42003_2018_75_MOESM2_ESM.docx]

**Description of Additional Supplementary Files**

File Name: Supplementary Data 1

Description: Data set for experimental cross-comparison

File Name: Supplementary Data 2

Description: Data set to compare phosphorylated and non-phosphorylated binding (Davis et al.).

File Name: Supplementary Data 3

Description: Data set of free energies: experimental IC 50 s, Prime, and FEP+.
